# Fertility test of mice (*Mus musculus*)

**DOI:** 10.21769/BioProtoc.5408

**Published:** 2025-08-05

**Authors:** Menglei Yang, Mengmeng Ming, Botao Yuan, Qinghua Shi, Baolu Shi

**Affiliations:** Center for Reproduction and Genetics, Department of Obstetrics and Gynecology, The First Affiliated Hospital of USTC, Hefei National Laboratory for Physical Sciences at Microscale, School of Basic Medical Sciences, Biomedical Sciences and Health Laboratory of Anhui Province, Institute of Health and Medicine, Hefei Comprehensive National Science Centre, Division of Life Sciences and Medicine, University of Science and Technology of China, Hefei, China

**Keywords:** Infertility, Gametogenesis, Spermatogenesis, Mouse, Fertility test

## Abstract

Infertility has emerged as a global health concern, impacting around 8%–12% of couples during their reproductive years. Due to limitations in obtaining human biological samples, mouse models have been widely used for investigating gene functions. Fertility assessment in mouse models is a critical component in reproductive biology for studying gene function and elucidating mechanisms of reproductive disorders. However, natural mating observation of mice may yield inconsistent results, especially in the absence of standard guidelines, prolonged experimental cycles, and operational complexity. This protocol establishes a comprehensive breeding strategy for evaluating murine fertility through systematic vaginal plug monitoring and litter size quantification within defined timeframes. Key steps include (1) standardized male–female pairing protocols, (2) daily vaginal plug inspection, and (3) longitudinal tracking of pregnancy outcomes. This protocol presents a straightforward and easily implementable protocol for mouse mating cage setup and statistical analysis, enabling reliable fertility assessment under natural breeding conditions.

Key features

• Standardized natural mating protocol combining vaginal plug monitoring (daily) and litter size tracking.

• Time-optimized workflow completes fertility phenotyping in 2 months.

• An easy and custom-constructed vaginal plug inspection tool for optimized vaginal plug inspection.

## Graphical overview


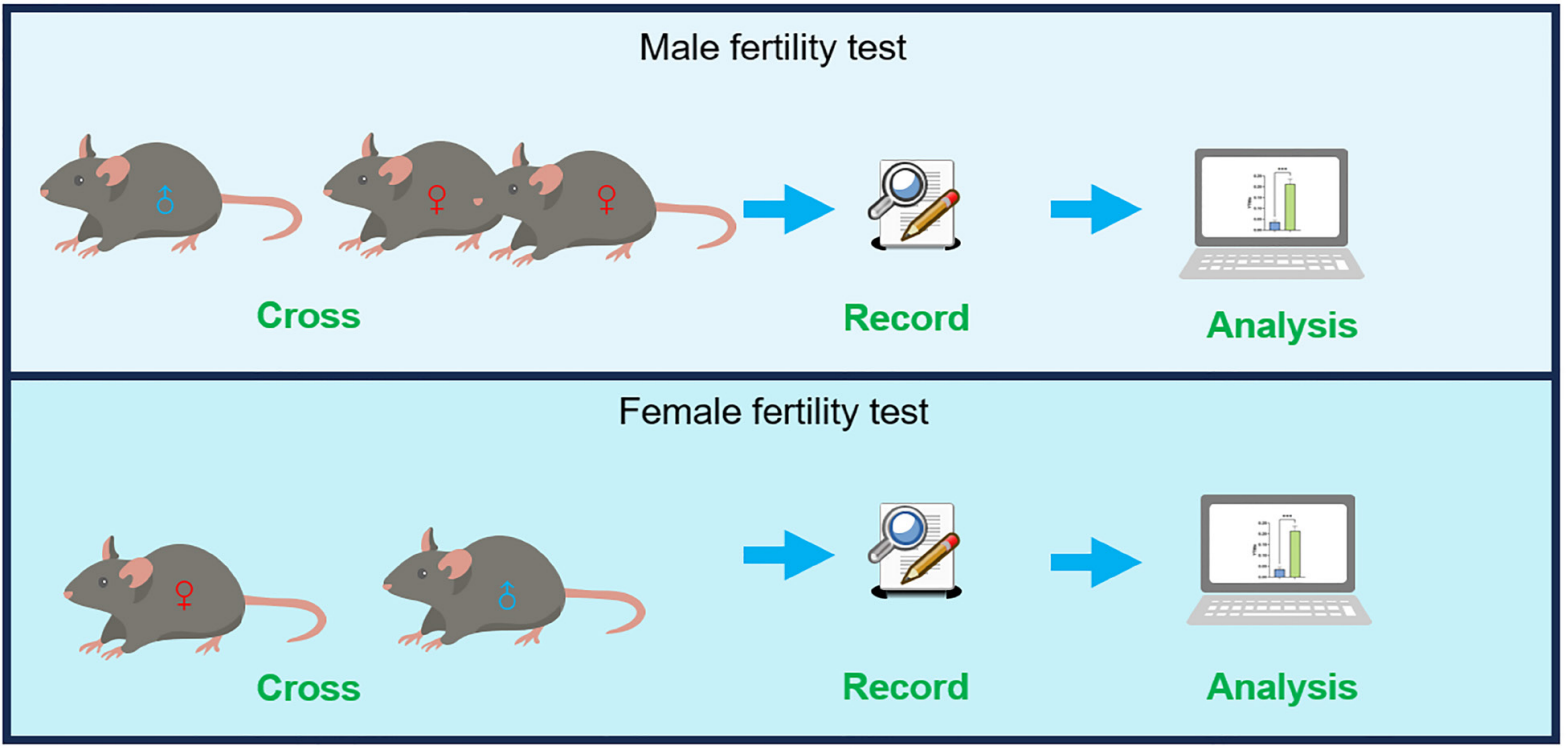


**Workflow overview of the fertility test of mice**

## Background

Infertility is classified as a medical condition characterized by the inability to attain a clinical pregnancy after 12 months or more of consistent, unprotected sexual intercourse, which affects approximately 48.5 million couples worldwide [1,2]. Due to a convergence of evolutionary conservation, experimental tractability, and short reproduction cycles, murine models serve as indispensable tools for deciphering the genetic basis of reproductive disorders [3,4]. Genetic editing technologies like CRISPR-Cas9 have advanced the study of the genetic basis of infertility by generating gene knockout or knock-in mouse models that mimic the mutations of infertile patients [5–7].

Fertility assessment forms the bedrock of both clinical and fundamental genetic research and offers value to clinical assisted reproductive technologies (ART) [8]. The function of a gene can be multifaceted, contributing to various organ systems. For researchers who lack expertise in reproductive medicine, an accurate evaluation of fertility necessitates the use of standardized references [9,10]. Nevertheless, the validity of fertility phenotyping remains critically dependent on standardized assessment methodologies [11]. Current challenges in natural mating observations include (1) subjective interpretation of copulatory plugs, (2) inconsistent mating durations leading to pseudopregnancy artifacts, and (3) lack of unified metrics for comparative fertility analysis across studies.

The current protocol establishes a detailed procedure for rodent fertility assessment by integrating standard cage-closing strategies and optimized methods for vaginal plug detection. This protocol offers a detailed description of the precautions to be taken during the caging process, providing a quick, easy, and accurate statistical and analytical strategy.

## Materials and reagents

**Biological materials**

1. C57BL/6J mice (Cyagen Biosciences, Suzhou, China)

**Reagents**

1. 75% ethanol (Sinopharm, catalog number: H-8017696)

2. 10% HCl (Sinopharm, catalog number: 7647-01-0)

3. Acidified ultrapure water (RO, 18.2 MΩ·cm), adjusted to pH 2.5–3.0 with 10% HCl

**Laboratory supplies**

1. Co-60 γ-ray sterilized breeding food (Co-Biotech, Nanjing, China)

2. Guard-type individually ventilated cage (Suzhou Fengshi Laboratory Animal Equipment Co., Ltd., China)

3. Laboratory animal wood shaving beddings (Co-Biotech, Nanjing, China)

4. Powder-free latex gloves (Haimen Yangzi Medical Equipment, Nantong, China)

5. Pipette tips; 1,000 μL (Biosharp, Hefei, China, catalog number: BS-1000-TR) and 10 μL (Biosharp, Hefei, China, catalog number: BS-10-TRS)

## Equipment

1. SPF-grade animal laboratory (Division of Life Sciences and Medicine, University of Science and Technology of China, Hefei, China)

## Software and datasets

1. Statistical Analysis Software Prism (GraphPad Software, San Diego, CA, USA, 10.1.2)

## Procedure

Laboratory rodent fertility is susceptible to environmental fluctuations. Uncontrolled variables (e.g., light cycles or stress from noise or handling) can disrupt hormonal regulation, directly impacting estrous cyclicity in females and libido in males. A lighting regimen of 14 h of light exposure and 10 h of darkness (14L:10D) (lights off at 18:00) synchronizes circadian-driven testosterone peaks in males, ensuring optimal mating vigor. This protocol provides step-by-step instructions for conducting a fertility evaluation.

**A. Mating competence test**

1. Acclimate males: House test males singly for ≥72 h before mating. Single housing of male mice prior to testing is critical to standardize their physiological and behavioral status.

*Note: A period of 72 h pre-test male isolation prevents social competition–induced testosterone suppression.*

**Figure 1.**
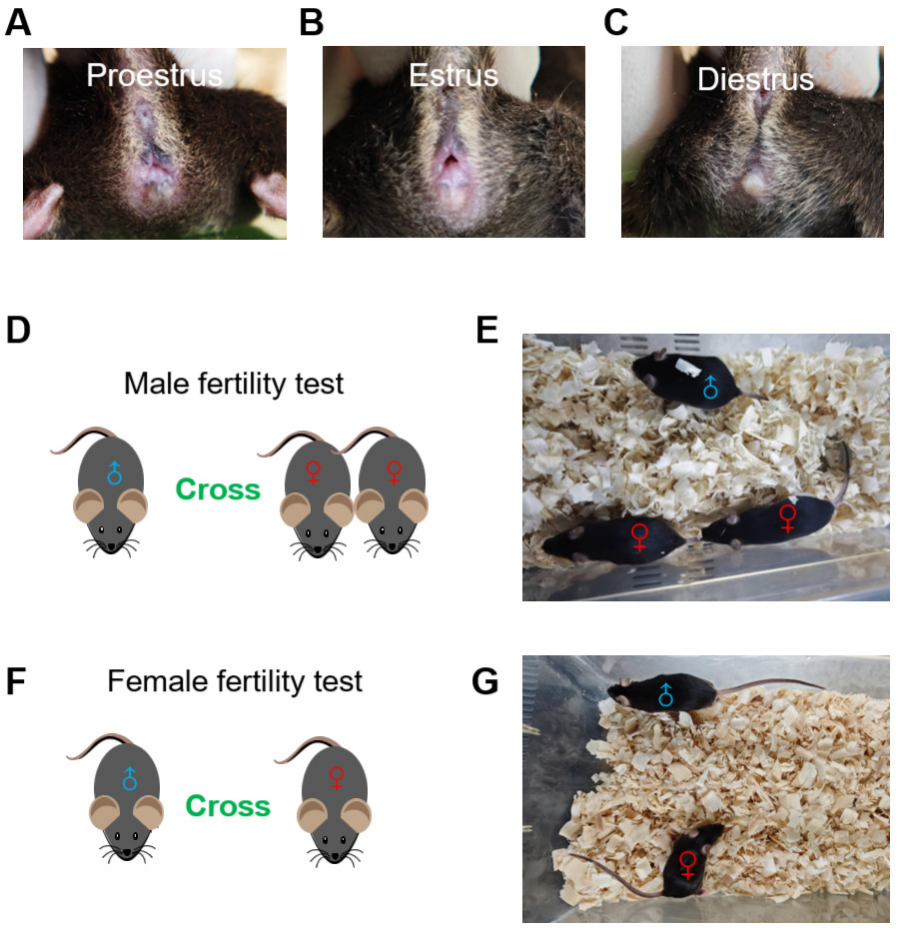
Strategy of the female and male mice fertility tests. (A) Representative image of a female mouse in proestrus. The vulva is slightly swollen and moist, with a partially open vaginal orifice. (B) Representative image of a female mouse in estrus. The vulva appears reddened and swollen, with a fully open, moist vaginal orifice. (C) Representative image of a female mouse in diestrus. The vaginal orifice is closed, and the vulva appears dry and pale. (D) For the male fertility test, transfer two females to each male’s cage. (E) Two females are crossed with one male mouse for the male fertility test. (F) For the female fertility test, transfer one male to each female’s cage. (G) The testing female mice are crossed with one male mouse for the female fertility test.

**Figure 2.**
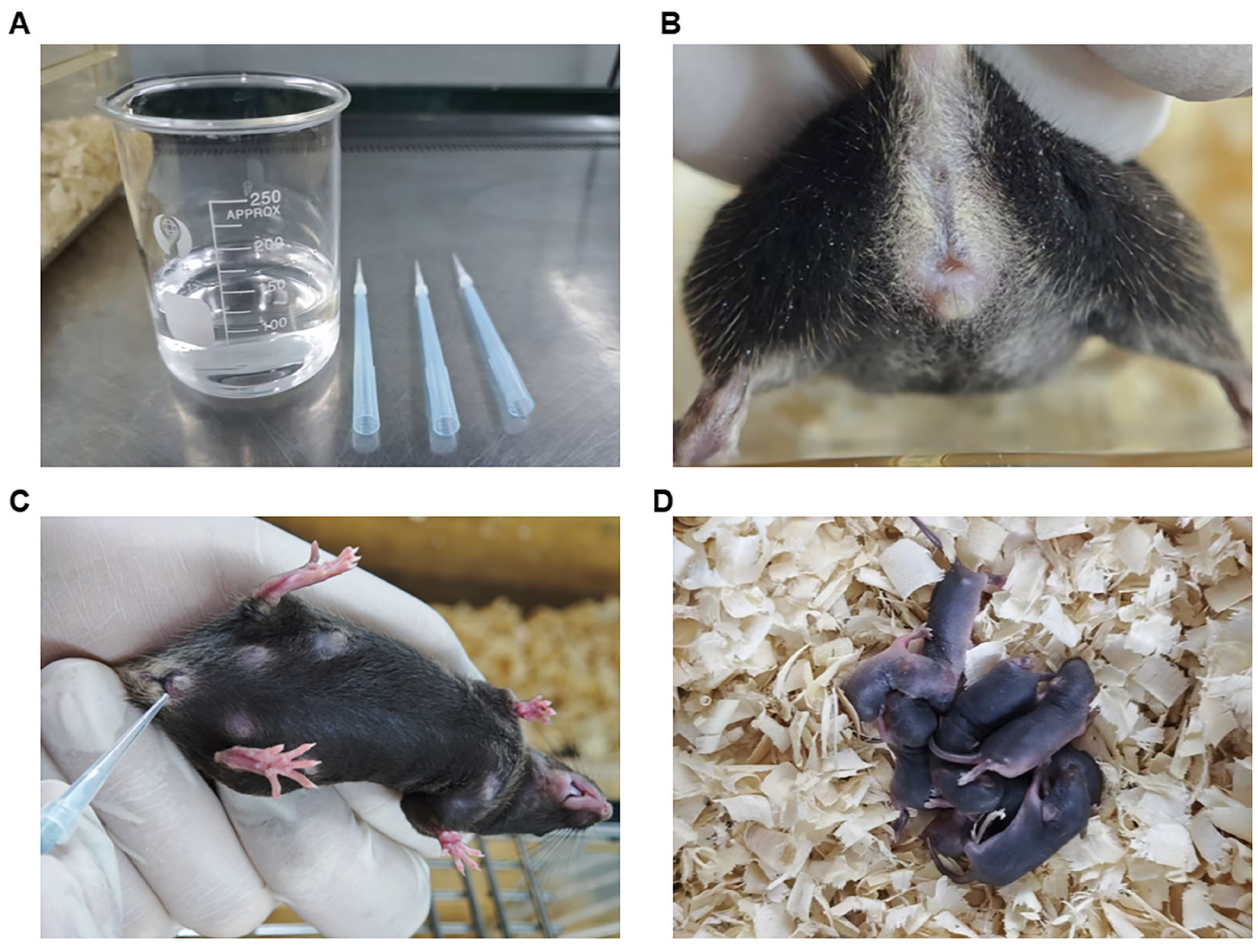
Inspection for detecting vaginal plugs. (A) Assemble a custom-constructed vaginal plug inspection tool by fixing a flat-trimmed 10 μL tip into a 1,000 μL tip box. (B) Observe the vaginal plugs by handling the female mice. (C) Gently insert the custom-constructed vaginal plug inspection tool into the vaginal orifice to check the vaginal plugs. (D) Count newborn pups.

2. Check female estrus: Females in proestrus or estrus should be preferentially selected for mating, as they exhibit high receptivity and pregnancy rates during these reproductive phases. Selection criteria include (1) proestrus females exhibiting a moderately swollen, moist vulva with a partially dilated vaginal orifice (Figure 1A), and (2) estrus females displaying full vulvar swelling with a patent, pinkish vaginal canal featuring distinct longitudinal folds on both dorsal and ventral labial surfaces (Figure 1B). Females in diestrus, characterized by complete vaginal closure, pale mucosal appearance, and absent vulvar edema (Figure 1C), should be excluded from mating trials.

3. Pair mating: At 16:00 (lights-off onset), for fertility testing of male mice, transfer two females to each male’s cage (Figure 1D, E). The purpose of using one male paired with two females (1M:2F) is to increase the potential pregnancy sample size per experiment and shorten the research cycle, as female mice exhibit considerable variability in hormonal status or external environment factors. For fertility testing of female mice, transfer one male to each female’s cage (Figure 1F, G).

*Note: It is recommended that both experimental and control mice be tested at the same time. For fertility testing of female mice, one female with one male is recommended due to the possibility of fights between adult males that may affect the state of the mice.*

4. Detect vaginal plugs: Assemble a custom-constructed vaginal plug inspection tool for detection by fixing a flat-trimmed 10 μL tip into a 1,000 μL tip box (Figure 2A). The next morning, first check the vaginal plugs by handling the female mice (Figure 2B) between 8:00 and 10:00. If vaginal plugs are not easily observed, double-check by gently inserting the custom-constructed vaginal plug inspection tool into the vaginal orifice (Figure 2C). Female mice with a vaginal plug show rotational resistance, while females without a vaginal plug allow smooth insertion. Record plug status daily for 5 days. If no plug is observed after 5 days, remove all females, rest the male for 48 h, and then replace with two new estrus-synchronized females for a second mating trial.

**B. Pup monitoring**

1. Housing conditions: Transfer females with vaginal plug to individual cages with ad libitum Co-60 γ-ray sterilized breeding diet/**acidified ultrapure** water.

*Note: Females usually give birth around 19–20 days after mid-bolt, so minimize disturbance to females and cage changes from around day 16 of gestation to 4 days after the birth of a litter.*

2. Pup records: After postnatal day 1 (P1), dip a powder-free latex glove into a bit of the bedding in the cage space to reduce odor interference and separate the mice along with some of the bedding for a quick count of the pups. Around P5–P7, conduct a detailed pup examination (Figure 2D).

*Note: Assign unique identification numbers if required for longitudinal studies.*

**Figure 3.**
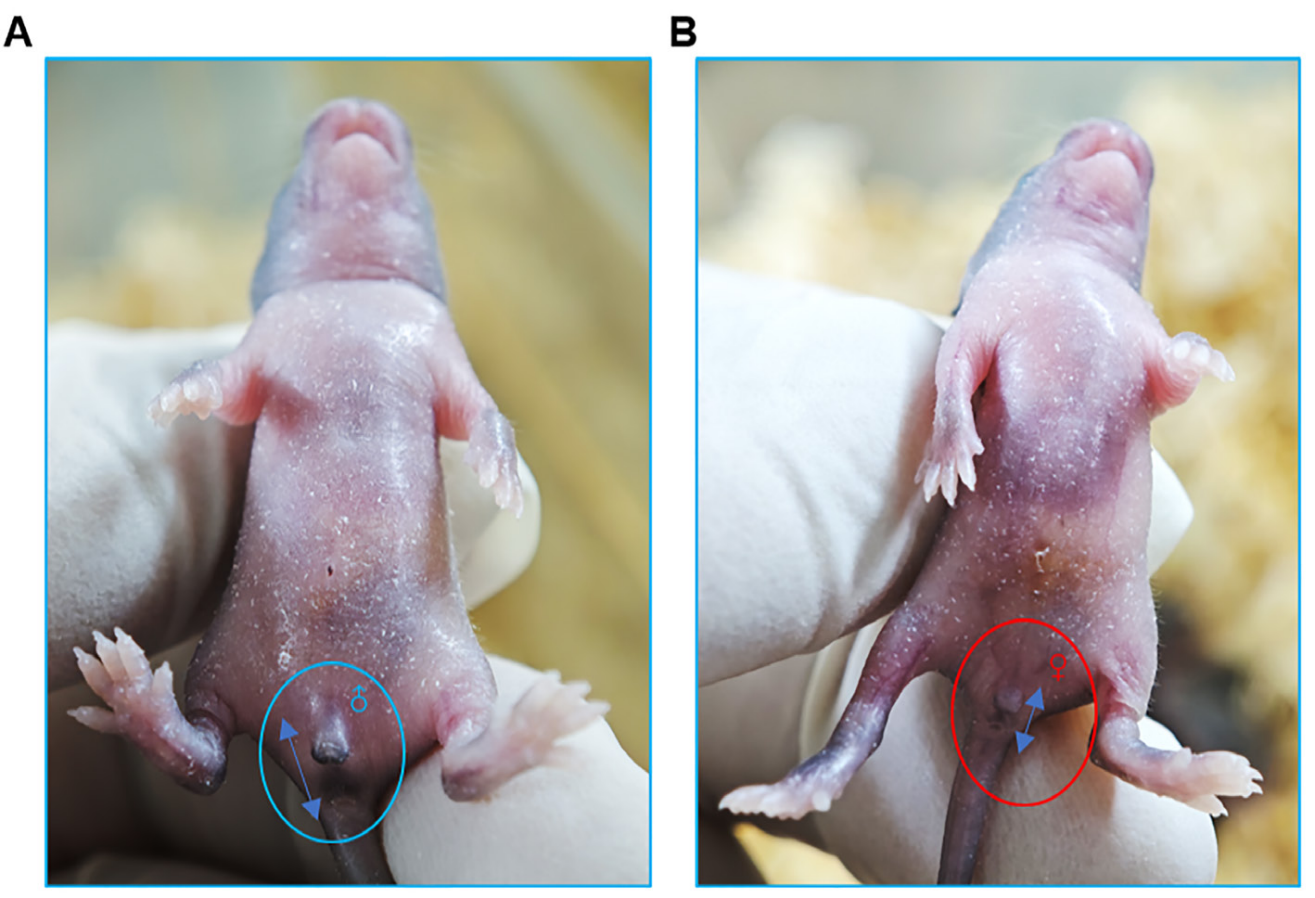
Distinguishing male and female newborn mice. (A) The genital protrusion of male mice is obvious, as indicated by the blue circle. Additionally, the anogenital distance (indicated by the bidirectional blue arrow) is greater, as indicated by the blue two-way arrow. (B) The genital protrusions of female mice are not obvious, as indicated by the red circle. Additionally, the anogenital distance is slightly smaller, as indicated by the blue two-way arrow.

3. Sex determination: Distinguishing male and female mice relies on quantitative measurement of the anogenital distance—the space between the genital papilla and the anus. Males exhibit a significantly greater anogenital distance than females due to in utero androgen exposure. You can also check the sex of the mice by observation of the genital papilla; males usually present more prominent genitals (Figure 3A, B). The number of pups and their sexes can be registered in a table similar to Table 1.

**Table 1.** Fertility test recording table

Record date	Genotype	Total born litter	♂ Count	♀ Count	Sex ratio (♂:♀)	Notes
05-23-2025	Wild type	7	4	3	1.3	
						
						
						
						

* ♂ denotes male mice; ♀ denotes female mice.

## Data analysis

1. For fertility analysis, the fertility test recording table data can be presented as the number of offspring per litter.

2. Values are calculated as mean ± standard error of mean (SEM) using GraphPad Software, 10.1.2.

3. Two groups of mice were compared with Student’s 2-tailed t-test for independent data. P < 0.05 was considered significant.

## Validation of protocol

This protocol has been used and validated in the following research article:

• Yang et al. [3]. Deficiency in DNAH12 causes male infertility by impairing DNAH1 and DNALI1 recruitment in humans and mice. *eLife* (Figure 4A, Figure 4—figure supplement 2A).

## General notes and troubleshooting

**General notes**

1. To narrow down the variables, it is recommended to use control group mice born in the same litter as the experimental group at the same time when performing the genetically modified mouse fertility test.

2. When checking for vaginal pessaries, a positive detection rate can be greatly improved with the aid of a homemade detector.

3. To rationalize the use of mice, mice born from fertility testing and parent mice can continue to be bred to reproduce the mouse population.

**Troubleshooting**

Problem 1: No vaginal plugs were observed after 5 days in the female fertility test.

Possible cause: Poor status of the male mice.

Solution: Replace the current group with male mice that have a history of impregnating female mice and producing offspring.

Problem 2: Reduced average litter size.

Possible cause: Cage changes or other disturbances occurred when the female was about to give birth.

Solution: Provide a suitable environment and reduce disturbances.
